# Altered Brain Fraction Amplitude of Low Frequency Fluctuation at Resting State in Patients With Early Left and Right Bell’s Palsy: Do They Have Differences?

**DOI:** 10.3389/fnins.2018.00797

**Published:** 2018-11-02

**Authors:** Xiaowei Han, Haimei Li, Xiaochun Wang, Yijiang Zhu, Tianbin Song, Lei Du, Shilong Sun, Runcai Guo, Jing Liu, Sumin Shi, Chao Fu, Wenwen Gao, Lu Zhang, Guolin Ma

**Affiliations:** ^1^Department of Radiology, China-Japan Friendship Hospital, Beijing, China; ^2^Graduate School of Peking Union Medical College, Beijing, China; ^3^Department of Radiology, Fu Xing Hospital, Capital Medical University, Beijing, China; ^4^Department of Radiology, First Clinical Medical College, Shanxi Medical University, Taiyuan, China; ^5^Department of Imaging, Anhui Provincial Hospital, Hefei, China; ^6^Department of Nuclear Medicine, Xuanwu Hospital, Beijing, China; ^7^Department of Science and Education, Shangluo Central Hospital, Shangluo, China

**Keywords:** fraction amplitude of low frequency fluctuation, amplitude of low frequency fluctuation, Bell’s palsy, rest-fMRI, Toronto Facial Grading System

## Abstract

**Purpose:** Bell’s palsy refers to acute idiopathic unilateral facial nerve palsy. It is a common disorder of the main motor pathway to the facial muscles. This study aimed to investigate the abnormal fraction amplitude of low frequency fluctuation (fALFF) of the brain in patients with early left and right Bell’s palsy.

**Materials and Methods:** Sixty-seven patients (left 33, right 34) and 37 age- and sex-matched healthy controls underwent resting-state functional magnetic resonance imaging (R-fMRI) examination. The fALFF values were measured from all subjects and were compared among the left palsy, right palsy, and control groups. Then, correlations between the Toronto Facial Grading System (TFGS) scores of the patients and the fALFF values of abnormal brain regions were analyzed.

**Results:** Significant group differences in fALFF values among the three groups were observed mainly in the cerebral cortical, subcortical, and deep gray matter regions. Compared with the right Bell’s palsy group, the left Bell’s palsy group showed significantly decreased fALFF values in the left temporal pole of the superior temporal gyrus (TPOsup), right supramarginal, left and right middle cingulate cortex (MCC), left superior frontal gyrus (SFG), and left precentral gyrus (PreCG), and increased fALFF values were observed in the right SFG and PreCG. Furthermore, altered fALFF values correlated positively with the TFGS scores in the left superior TPO, bilateral MCC, and right PreCG, and correlated negatively with the TFGS scores in the right SFG of the left Bell’s palsy group. Altered fALFF values correlated positively with the TFGS scores in the bilateral MCC and right PreCG and correlated negatively with the TFGS scores in the left superior TPO and SFG of the right Bell’s palsy group.

**Conclusion:** Regulatory mechanisms seem to differ between patients with left and right early Bell’s palsy. The severity of the disease is associated with these functional alterations.

## Introduction

Bell’s palsy refers to acute idiopathic unilateral facial nerve palsy. It is a common disorder of the main motor pathway to the facial muscles (Holland and Weiner, 2004; Basić-Kes et al., 2013). The facial nerve is involved in controlling facial symmetry, facial expressions, movements, and other functions (Spencer and Irving, 2016; Hussain et al., 2017). Thus, Bell’s palsy can result in considerable psychological impact on patients. Extensive research on brain function reorganization in this condition is needed to understand the mechanisms of functional integration within the cerebral cortex (Gupta et al., 2013; Portelinha et al., 2014). Furthermore, investigation of patients with different-sided Bell’s palsy at the early stage could facilitate insight into differences in the mechanisms involved in functional integration and may provide a basis for implementation of suitable treatment as early as possible.

As a new advanced technique, functional magnetic resonance imaging (fMRI) has been widely used in evaluating many diseases including Bell’s palsy (Hillman, 2014; Buchbinder, 2016). These previous studies were mainly task-based fMRI studies, and found that some cortical functional activities are interrupted at the early stages in patients with Bell’s palsy. Motor integration is increased, and functional motor integration mainly occurs in the hemisphere contralateral to the paralyzed side (Barkhof et al., 2014; He et al., 2014; Zacà et al., 2014). With the gradual recovery of facial nerve function, the functional activities in the related brain regions also gradually recover to the normal level (Rijntjes et al., 1997; Bitter et al., 2011; Wu et al., 2014). fMRI in the resting state (R-fMRI) is less demanding for patient cooperation than that during complex activation tasks (Buxton, 2013; Conklin et al., 2014; Fovet et al., 2015). However, research using R-fMRI in patients with Bell’s palsy is still in its infancy (Klingner et al., 2011, 2012). R-fMRI provides useful information for exploring functional brain changes and interpretation of task-based fMRI. Slow fluctuation in activity is a fundamental feature of the resting brain (Smit et al., 2010; Buendia et al., 2016), and is also called low frequency oscillation (LFO).

The amplitude of low-frequency fluctuation (ALFF) is one of the quantitative methods that is often used in R-fMRI (Zang et al., 2007). ALFF is defined as the total power within the low frequency range (from 0.01 to 0.1 Hz) and indicates the strength of LFOs (Zang et al., 2007). The fractional ALFF (fALFF) is a methodological improvement of ALFF; it is defined as the power within the low-frequency range divided by the total power in the entire detectable frequency range. Thus, fALFF indicates the relative contribution of LFOs to the whole frequency range (Zou et al., 2008). Both ALFF and fALFF can reflect the spontaneous activity of the brain from the perspective of brain energy metabolism (Orringer et al., 2012; Buckner et al., 2013). fALFF is more sensitive and specific than ALFF at low frequencies and can more accurately reflect the strength of spontaneous activity in the brain (Zou et al., 2008; Jing et al., 2013). Early studies using fALFF found that patients with Bell’s palsy showed significant fALFF decreases in the contralateral primary somatosensory cortex and the primary motor cortex. However, for patients with early Bell’s palsy, it is unclear whether there are bilateral differences in abnormal functional activities. Therefore, we used fALFF to evaluate the functional alterations in patients with early Bell’s palsy in order to identify abnormal brain functional activities and to identify the difference in altered brain regions between patients with left and right Bell’s palsy.

In this study, we hypothesized that patients with left or right Bell’s palsy at the early stage would have abnormal fALFF in some brain areas. Moreover, since the Toronto Facial Grading System (TFGS) (Ahrens et al., 1999; Kayhan et al., 2000) plays an important role in assessment of facial nerve damage and inability, we also hypothesized that the TFGS would be associated with fALFF alterations. To test our hypothesis, the resting-state fALFF of patients with early Bell’s palsy were investigated and compared with those in healthy controls. Additionally, we analyzed the correlation between fALFF values and clinical severities in the left and right palsy groups.

## Materials and Methods

### Subjects

Sixty-seven patients were enrolled from December 2015 to May 2017 in our hospital. Bell’s palsy was diagnosed by two experienced neurologists using the TFGS. The patients were divided into the left facial paralysis group (33 cases, 12 men and 21 women, age 48.11 ± 13.27 years) and the right facial paralysis group (34 cases, 13 men and 21 women, age 47.27 ± 12.96 years). The inclusion criteria were: (1) adult patients with first-ever idiopathic unilateral facial nerve palsy (the diagnosis was confirmed by the two neurologists), (2) onset time of 2-7 days, (3) no other brain and psychiatric diseases, no use of psychotropic drugs; and (4) being right-handed. Thirty-seven healthy controls (14 men and 23 women, age 46.05 ± 13.65 years) were recruited from postgraduate students, hospital staff, and by advertisement. The inclusion criteria for healthy controls were: (1) absence of systemic diseases and neurological symptoms and signs; (2) absence of a family history of neurological diseases; (3) normal head MRI examination; (4) TFGS scores of 100 points; (5) being right-handed. The study was approved by the Ethics Committee of our hospital, and written informed consent was obtained from each participant.

### MRI Data Acquisition

Participants were instructed to rest in a supine position with eyes closed and to breathe calmly, with the head fixed to minimize head movement. Participants were asked not to think and to remain awake, Rubber earplugs were used to reduce noise. A 3.0 T MR scanner (GE Healthcare, Discovery MR750, Milwaukee, WI, United States) with a supporting head quadrature coil was used for MRI. The scan protocol included: (1) scout images; (2) T2-weighted imaging (T2WI); (3) resting state blood oxygen-level dependent-fMRI with single-shot gradient recalled echo-planar imaging sequence [parameters were: slice thickness = 3.5 mm, slice spacing = 0.7 mm, repetition time (TR) = 2000 ms, echo time (TE) = 30 ms, flip angle = 90°, matrix = 64 × 64, field of view (FOV) = 240 mm × 240 mm, and number of excitations (NEX) = 1, 34 slices and 240 phases]; (4) 3D T1-weighted imaging (T1WI) with three-dimensional fast spoiled gradient-echo sequences (3D FSPGR) (Scanning parameters: slice thickness = 1.0 mm, TR = 6.7 ms, TE = Min Full, acquisition matrix = 256 × 256, FOV = 256 mm × 256 mm, and NEX = 1).

### Data Pre-processing and fALFF Calculation

Data were preprocessed with the Data Processing Assistant for Resting-State fMRI (DPARSF) software (Yan et al., 2016). The procedure involved conversion of the NIFTI format, removing the first 10 time points, slice timing, head motion correction (standard: 2.0 mm or 2°) and movement parameter acquisition, spatial normalization with T1 anatomical image unified segmentation, spatial smoothing (full-width at half-maximum set as 4), and linear drift removal. Then, the white matter and cerebrospinal fluid (CSF) signals were eliminated with covariate regression analysis. Finally, fALFF was calculated and band-pass filtering (0.01–0.08 Hz) was performed to remove the effects of low-frequency drift and high-frequency noise.

### Statistical Analysis

SPSS (version 19.0, Chicago, IL, United States) was utilized to analyze demographic and clinical data. One-way analysis of variance (ANOVA) was employed to compare the age and education level, and a chi-square test was used to compare sex among the three groups. Independent two-sample *t*-tests were applied to compare the illness duration and TFGS scores between left and right Bell’s palsy groups. One-way analysis of covariance (ANCOVA) was conducted to examine the difference in fALFF among the three groups. Age, sex, education level, and head movement parameters were incorporated as covariates. *Post hoc* *t*-tests were conducted to identify differences between every pair of groups. False discovery rate (FDR) correction was used for multiple comparison correction in the voxel-based fMRI statistical map analyses, with a threshold of 0.05 (Song et al., 2017).

Every brain region showing statistically significant differences between left and right palsy groups was saved as a cluster mask; then, the masks were used to extract the fALFF values using the Extract ROI tool of DPARSF. Pearson’s correlation was used to analyze the difference in TFGS score between the patients and their corresponding fALFF values in these brain regions further, by using SPSS software. Statistical significance was set at *P* < 0.05.

## Results

### Clinical Data

There were no statistically significant differences in sex, age, or education level between patients with Bell’s palsy and healthy controls (*P* > 0.05). The duration from onset to examination was 0 days and the TFGS scores were 100 in healthy controls. Disease duration among patients was comparable in the left (mean ± standard deviation: 4.83 ± 2.14 days) and right (mean ± standard deviation: 4.42 ± 2.56 days) facial palsy groups (*P* > 0.05). There was no significant difference in TFGS scores between the left and right Bell’s palsy groups (*P* > 0.05). However, the TFGS scores of the left and right Bell’s palsy groups were significantly lower than those of the healthy controls (Table 1).

**Table 1:** Demographic and clinical data of the study population.

Groups	Gender (M/F)	Age (years)	Education (years)	Duration (days)	TFGS (scores)
Left Bell’s palsy	12/21	48.11 ± 13.27	11.76 ± 3.15	4.83 ± 2.14	19.24 ± 16.09
Right Bell’s palsy	13/21	47.27 ± 12.96	12.38 ± 2.85	4.52 ± 2.46	17.55 ± 16.22
Healthy controls	14/23	46.05 ± 13.65	14.13 ± 2.33	0	100.00
*P*-value	0.48	0.12	0.09	0.37^∗^	0.28^∗^


*Values are expressed as mean ± SD. *TFGS*, Toronto Facial Grading System. ^∗^*P*-value calculated between left and right Bell’s palsy with independent two-sample *t*-test.*

### Group Differences in fALFF Value

**FIGURE 1:**
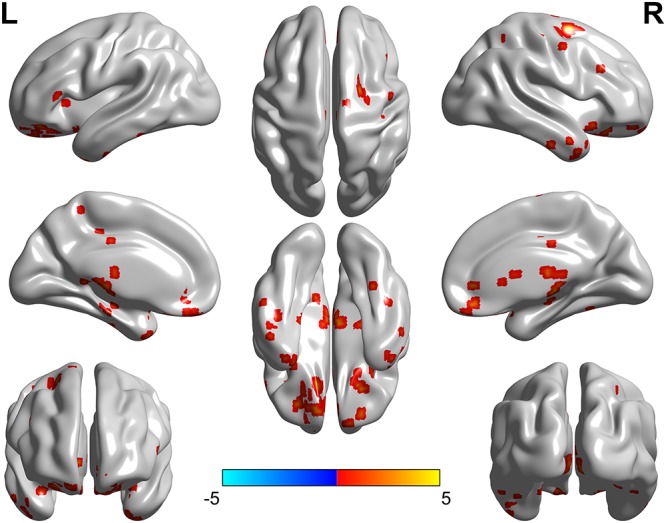
Brain regions showed fALFF differences among the three groups (left, right Bell’s palsy groups and control group). The abnormal regions were observed mainly in the cerebral cortical, subcortical and deep gray matter regions.

Significant group differences in fALFF among patient and control groups were observed mainly in the cerebral cortical, subcortical and deep gray matter regions (Figure 1).

**FIGURE 2:**
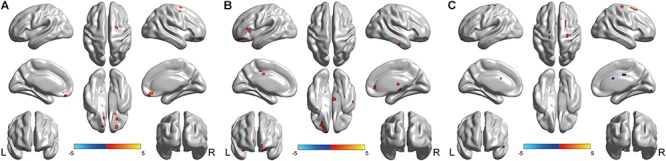
Brain regions showed fALFF differences between every pair of groups with *post hoc* *t*-tests. **(A)** Brain regions showed fALFF differences between left Bell’s palsy and control groups. Compared with control group, left Bell’s palsy group showed significantly increased fALFF values in the bilateral rectus, left ORBsup, right ORBmid, right SFG, right PreCG, and right putamen. **(B)** Brain regions showed fALFF differences between right Bell’s palsy and control groups. Compared with control group, right Bell’s palsy group showed significantly increased fALFF values in the right ITG, right ORBinf, left ORBmid, left ORBsup, left TPOsup, left caudate, bilateral ACC, left IFGtriang, bilateral MCC, and left IPL. **(C)** Brain regions showed fALFF differences between left and right Bell’s palsy groups. Compared with right Bell’s palsy group, left Bell’s palsy group showed significantly decreased fALFF values in the left TPOsup, right SMG, bilateral MCC, left SFG, and left PreCG; the left Bell’s palsy showed significantly increased fALFF values were the right SFG and PreCG. TPOsup, superior temporal gyrus in the temporal pole; SMG, supramarginal; MCC, middle cingulate cortex; SFG, superior frontal gyrus; PreCG, precentral gyrus; ORBsup, orbital part of superior frontal gyrus; ORBmid, orbital part of middle frontal gyrus; ITG, inferior temporal gyrus; ORBinf, orbital part of Inferior frontal gyrus; ACC, anterior cingulate cortex; IFGtriang, triangular part of inferior frontal gyrus; IPL, left inferior parietal lobule.

In the left Bell’s palsy group, the brain regions that showed significantly higher fALFF values than those in the healthy control group were the bilateral rectus, left orbital part of the superior frontal gyrus (ORBsup), right orbital part of the middle frontal gyrus (ORBmid), right superior frontal gyrus (SFG), right precentral gyrus (PreCG), and right putamen (Table 2 and Figure 2A).

**Table 2:** Brain regions showing group fALFF differences in *Post hoc t-tests* analysis.

Brain regions	BA	Peak MNI Coordinates (mm) (x, y, z)	*t*-value
Left vs. healthy controls			
Rectus.L	11	(-9, 39, -18)	2.92
Rectus.R	11	(6, 42, -15)	2.72
ORBsup.L	11	(-12, 48, -21)	2.83
ORBmid.R	11	(24, 48, -15)	2.73
SFG.R	6	(24, 0, 63)	2.87
PreCG.R	6	(24, -6, 48)	3.03
Putamen.R	/	(24, -6, 12)	2.82
Right vs. healthy controls			
ITG.R	20	(57, -15, -30)	2.46
ORBinf.R	38	(33, 15, -21)	2.56
ORBmid.L	11	(-18, 54, -15)	3.32
ORBsup.L	11	(-12, 66, -12)	2.72
TPOsup.L	38	(-54, 15, -12)	2.61
Caudate.L	25	(-9, 15, -6)	2.54
ACC.L	11	(-11, 39, -3)	2.78
ACC.R	11	(9, 42, 0)	2.44
IFGtriang.L	45	(-54, 24, 3)	2.58
MCC.L	23	(-18, -30, 39)	3.11
MCC.R	23	(9, -12, 36)	2.57
IPL.L	40	(-30, -48, 45)	2.68
Left vs. right palsy			
TPOsup.L	38	(-51, 9, -9)	-2.19
SMG.R	48	(65, -38, 25)	-2.21
MCC.L	23	(0, -18, 33)	-2.48
MCC.R	23	(2, -18, 34)	-2.36
SFG.L	6	(-15, 12, 54)	-2.79
SFG.R	8	(24, 12, 60)	2.45
PreCG.L	6	(-24, -12, 75)	-2.51
PreCG.R	6	(36, -18, 69)	2.57


*BA, Brodmann area; MNI, Montreal Neurologic Institute; TPOsup.L, left temporal pole of superior temporal gyrus; SMG.R, right supramarginal; MCC.L, left middle cingulate cortex; MCC.R, right middle cingulate cortex; SFG.L, left superior frontal gyrus; SFG.R, right superior frontal gyrus; PreCG.L, left precentral gyrus; PreCG.R, right precentral gyrus; Rectus.L, left rectus; Rectus.R, right rectus; ORBsup.L, left orbital part of superior frontal gyrus; ORBmid.R, right orbital part of middle frontal gyrus; Putamen.R, right putamen; ITG.R, right inferior temporal gyrus; ORBinf.R, right orbital part of Inferior frontal gyrus; Caudate.L, left caudate; ACC.L, left anterior cingulate cortex; ACC.R, right anterior cingulate cortex; IFGtriang.L, left triangular part of inferior frontal gyrus; IPL.L, left inferior parietal lobule.*

In the right Bell’s palsy group, the brain regions that showed significantly higher fALFF values than those in the healthy control group were the right inferior temporal gyrus (ITG), right orbital part of inferior frontal gyrus (ORBinf), left orbital part of the middle frontal gyrus, left orbital part of the superior frontal gyrus, left temporal pole of the superior temporal gyrus (TPOsup), left caudate, left and right anterior cingulate cortex (ACC), left triangular part of the inferior frontal gyrus (IFGtriang), bilateral middle cingulate cortex (MCC), and left inferior parietal gyrus (IPL) (Table 2 and Figure 2B).

Compared with the right Bell’s palsy group, the left Bell’s palsy group showed significantly decreased fALFF values in the left temporal pole of the superior temporal gyrus, right supramarginal (SMG), left and right middle cingulate cortex, left superior frontal gyrus, and left precentral gyrus, and significantly increased fALFF values in the right superior frontal gyrus and precentral gyrus (Table 2 and Figure 2C).

### Correlations Between TFGS Scores and Altered fALFF Values

**FIGURE 3:**
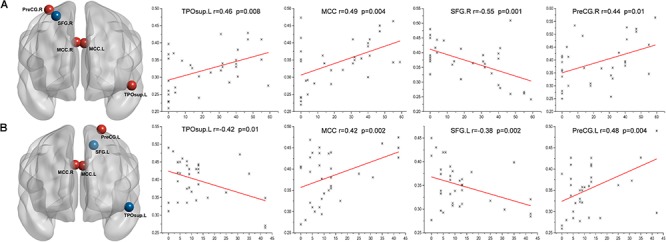
Correlations between the TFGS scores of Bell’s palsy patients and their corresponding altered fALFF values of abnormal brain regions. **(A)** In the left Bell’s palsy group, the brain regions showing a positive correlation were the left TPOsup, bilateral MCC, and right PreCG; the brain region indicating a negative correlation was the right SFG. **(B)** In the right Bell’s palsy group, the brain regions showing a positive correlation were the bilateral MCC and left PreCG; the brain regions showing a negative correlation were the left TPOsup and SFG. TPOsup, superior temporal gyrus in the temporal pole; MCC, middle cingulate cortex; PreCG, precentral gyrus; SFG, superior frontal gyrus.

In the left Bell’s palsy group, the fALFF values showed positive correlations with the TFGS scores in the left superior TPO (*P* < 0.05), left and right MCC (*P* < 0.01), and the right PreCG (*P* < 0.05). The only brain region indicating a negative correlation between fALFF values and TFGS score was the right SFG (*P* < 0.05) (Table 3 and Figure 3A).

**Table 3:** Brain regions showed correlation between fALFF value and TFGS score.

	Two brain regions	*r*- values	*P*-values
Left facial palsy	TPOsup.L	0.46	0.008
	MCC.L & MCC.R	0.49	0.004
	SFG.R	-0.55	0.001
	PreCG.R	0.44	0.01
Right facial palsy	TPOsup.L	-0.42	0.01
	MCC.L & MCC.R	0.42	0.02
	SFG.L	-0.38	0.02
	PreCG.L	0.48	0.04


*BA, Brodmann area; MNI, Montreal Neurologic Institute; TPOsup.L, left temporal pole of superior temporal gyrus; SMG.R, right supramarginal; MCC.L, left middle cingulate cortex; MCC.R, right middle cingulate cortex; SFG.L, left superior frontal gyrus; SFG.R, right superior frontal gyrus; PreCG.L, left precentral gyrus; PreCG.R, right precentral gyrus.*

In the right Bell’s palsy group, the fALFF values showed positive correlations with the TFGS scores in the left and right MCC (*P* < 0.05) and the right PreCG (*P* < 0.05). The fALFF values showed negative correlations with the TFGS scores in the left superior TPO (*P* < 0.05) and the left SFG (*P* < 0.05) (Table 3 and Figure 3B).

## Discussion

Pathologically, peripheral facial palsy is caused by simple peripheral nerve efferent dysfunction, in which the cranial nerve nuclei cannot control the movement of facial muscles but can still receive sensory information (Roob et al., 1999; Burmeister et al., 2011; Klingner et al., 2014). One of the most significant features of the human brain is its ability to adapt to new environments and accept outside information to remodel functions of the cerebral cortex (Brändle et al., 1996; Klingner et al., 2011).

This study found that in both left and right Bell’s palsy groups, the prefrontal cortex (Rectus, ORB, and IFG) and bilateral cingulate cortex showed abnormal fALFF when compared with the healthy control group. These brain regions are associated with emotion processing and are involved in depression, anxiety, and other negative emotional states (Schaefer et al., 2006; Drevets et al., 2008; Mohamed et al., 2014). Compared with the healthy control group, the left Bell’s palsy group showed increased fALFF values in the right SFG, PreCG and putamen which are involved in the remodeling of the motor network (Goldberg et al., 2006). The right palsy group exhibited abnormal fALFF values in the left caudate nucleus, which is related to motor function (Jiji et al., 2013). In addition, only the right palsy group showed increased fALFF values in the right ITG, left TPOsup, and IPL which are associated with sensory transmission and emotion perception (Bigler et al., 2007; Jou et al., 2010). The regions related to sensory transmission and emotion perception differed between the patient groups in this study. These differences imply that the bilaterally different reintegration mechanisms probably derive from asymmetrical compensation for the abnormal functions in the left and right hemispheres (Liu et al., 2014).

Compared with the right Bell’s palsy group, the left Bell’s palsy group showed increased fALFF values in the contralateral SFG and PreG and decreased fALFF values in the ipsilateral SFG and PreCG. These brain regions are involved in remodeling of the motor network (Goldberg et al., 2006). In peripheral facial palsy, the major facial motor nerve pathways are damaged, resulting in reintegration of the motor network to compensate for lost motor function (Grefkes et al., 2010). The SFG is related to self-awareness and laughter and is an important gray matter structure that usually demonstrated abnormalities in patients with Bell’s palsy in the previous studies (Goldberg et al., 2006; He et al., 2014; Wu et al., 2014). We assume that the dysfunction of facial muscles in the patients might, at least in part, be the cause of change in the functional activity of contralateral SFG. This finding is in line with previous finding in the literature that state that active reintegration of motor function occurs in the contralateral hemisphere in patients with Bell’s palsy (Liu et al., 2014). The contralateral hemisphere plays an important role in motor processes and inhibitory control of action in patients with Bell’s palsy (Jiji et al., 2013).

Compared with the right Bell’s palsy group, the left Bell’s palsy group showed decreased fALFF values in the left TPOsup, right SMG, and left and right MCC. The superior temporal gyrus has been reported to be involved in language processing and social perception (Bigler et al., 2007; Jou et al., 2010). The supramarginal gyrus forms part of the somatosensory association cortex, and is involved in the perception of the emotions, conveyed by postures and gestures of other people (Hartwigsen et al., 2010). The differences in fALFF in these regions demonstrated that emotion perception and somatosensory sense in patients with left facial palsy show more marked alterations than those in patients with right facial palsy. It is also interesting that the left and right middle cingulate cortex demonstrated higher fALFF values in patients with left facial palsy than those in patients with right facial palsy. The cingulate cortex is an integral part of the limbic system, which is involved in emotion formation and processing, as well as memory (Stanislav et al., 2013). These differences probably reflect different mechanisms of functional integration of emotion between patients with early left and right Bell’s palsy (Schaefer et al., 2006).

Few studies have reported a correlation between fALFF values and TFGS scores in patients with Bell’s palsy. In the present study, the brain regions showing a positive correlation with TFGS scores in patients with left Bell’s palsy were the left TPOsup, left and right MCC, and right PreCG, while those in patients with right Bell’s palsy were the bilateral MCC, and right PreCG; these brain regions were mainly associated with motor and emotion information processing functions. These findings suggest that facial muscle recovery benefits from motor and sensory regulation in patients with Bell’s palsy (Lorch and Teach, 2010). However, it is still not clear whether increased motor training and maintenance of the total amount of sensory information in the early stages are helpful for recovery (Klingner et al., 2014). The bilateral MCC in the left and right Bell’s palsy group correlated positively with the TFGS scores. These findings indicate that the MCC is involved, to some degree, in linking sensorimotor outcomes to emotion (Drevets et al., 2008). In the present study, the right SFG showed a negative correlation with TFGS scores in patients with left Bell’s palsy, while such correlations were seen in the left TPOsup and the right SFG in patients with right Bell’s palsy. These regions are mainly related to emotion perception and self-awareness. Negative emotions derive from abnormal facial expressions which can cause patients with Bell’s palsy to be more self-aware (Lorch and Teach, 2010). Thus, the psychological state of negative emotion and self-awareness can be a reflection, at least in part, of clinical symptoms in patients with early Bell’s palsy (Goldberg et al., 2006).

This study had some limitations. The sample size was relatively small. The findings of this study need to be confirmed in studies with larger study populations. Moreover, since we did not conduct follow-up or dynamic observations, our study only focused on the fALFF changes in patients at the acute stage. In future studies, the number of cases should be increased and patients should be grouped by disease stage. Extensive further investigations are needed to determine the full significance of fALFF alterations in these patients.

## Conclusion

In summary, this study provided significant evidence for abnormal brain activity between patients with early left and right Bell’s palsy. In addition, the severities of the disease were closely associated with abnormal fALFF values in certain brain regions. We also observed differences in abnormal fALFF values in patients affected on different sides, indicating that the reintegration mechanisms in patients with left and right facial palsy may differ. Further studies are needed to elucidate the exact underlying mechanisms and meaning of altered fALFF values in the brains of patients with Bell’s palsy.

## Author Contributions

XH, HL, and XW contributed equally, they acquired, analyzed, and explained data, drafted the manuscript and revised it. XW, YZ, and TS analyzed and explained the data. LD, SS, RG, JL, SS, CF, and WG designed scans and acquired the data. LZ and GM revised the manuscript for extremely important intellectual content.

## Conflict of Interest Statement

The authors declare that the research was conducted in the absence of any commercial or financial relationships that could be construed as a potential conflict of interest.
